# Analysis of thermally driven structural changes, genome release, disassembly, and aggregation of recombinant AAV by CDMS

**DOI:** 10.1016/j.omtm.2022.10.008

**Published:** 2022-10-14

**Authors:** Lauren F. Barnes, Benjamin E. Draper, Martin F. Jarrold

**Affiliations:** 1 Chemistry Department, Indiana University, 800 E Kirkwood Avenue, Bloomington, IN 47405, USA; 2 Megadalton Solutions, Inc., 3750 E Bluebird Ln, Bloomington, IN 47401, USA

**Keywords:** adeno-associated virus, AAV, rAAV, charge detection mass spectrometry, CDMS, mass spectrometry, genome release, thermal stability

## Abstract

Charge detection mass spectrometry (CDMS) was used to analyze recombinant adeno-associated virus serotype 8 (rAAV8) vectors after incubation at elevated temperatures. rAAV8 vectors with a range of genomes of interest (GOIs) from 2.22 to 4.84 kb were investigated. For the shorter GOIs, GOI release occurred at surprisingly low temperatures (15 min at 45°C for cytomegalovirus [CMV]-GFP). The released DNA and intermediates with the GOI extruded from the capsid were detected. The temperature required to release the short GOIs is well below the 65°C incubation temperature required to disassemble the empty rAAV8 capsid. The temperature for GOI release increased with its GOI length. With the longer GOIs, the GOI stabilized the capsid so that it remained intact under conditions that would disassemble the empty particle. After incubation at 65°C, the main species in the CDMS mass distributions for the longer GOIs was the vector with the GOI. However, for GOIs longer than the wild-type genome (∼4.7 kb), the stability diminished, and genome release occurred at a lower temperature. Heterogeneous DNA fragments from the host cells or plasmids is released at a lower temperature than the longer GOIs, suggesting that the GOIs have a feature that resists early release.

## Graphical abstract

**Figure:**
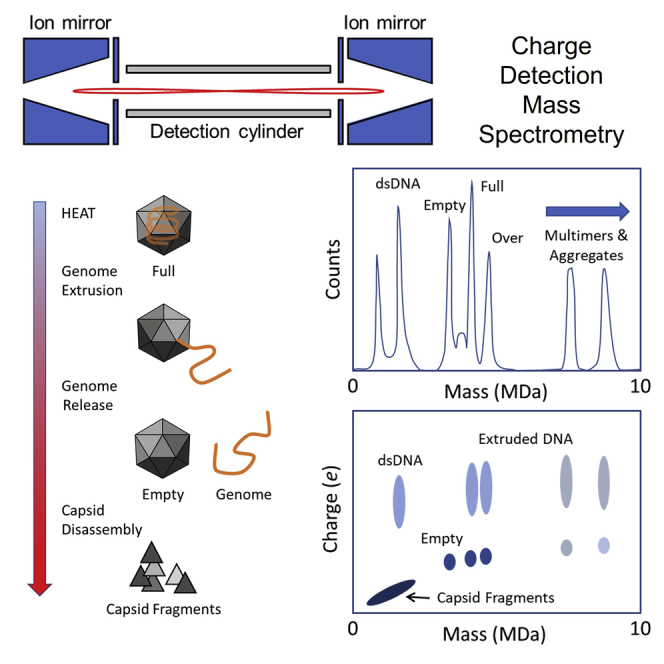

## Teaser

Charge detection mass spectrometry was used to analyze rAAV8 vectors after incubation. Particles with extruding DNA and released genome were detected. For shorter genomes, release occurred at low temperature, less than required for empty capsid disassembly. Longer genomes stabilized the capsid, and it remained intact when the empty capsid disassembles.

## Introduction

Recombinant adeno-associated virus (rAAV) is a leading gene therapy vector for monogenetic diseases. There are currently two FDA-approved therapies and hundreds more in clinical trials.^1^^,^^2^ Its favorable attributes include low immunotoxicity and wide cell trophism. AAV, a member of the parvovirus family, is one of the smallest and simplest viruses. It is around 25 nm in diameter and consists of a capsid surrounding a packaged genome. The capsid contains 60 proteins, a mixture of the viral proteins VP1, VP2, and VP3, arranged in pseudo icosahedral symmetry.^3^, ^4^, ^5^ The three capsid proteins are generated from overlapping reading frames in the *cap* gene. They all contain a common VP3 sequence at the C terminus. VP2 is missing residues 1–137 of the VP1 sequence, and VP3 is missing an additional 66 residues (i.e., 1–203) of the VP1 sequence. The VP1 unique region contains a motif homologous to a phospholipase A2 domain. The VP1 unique region and VP1/VP2 common regions are initially internalized,^6^^,^^7^ but at some point during infection, a conformational change promotes their exposure.^6^^,^^8^ It is thought that each site on the icosahedral *T* = 1 capsid is populated randomly by a VP1, VP2, or VP3 in ratios that mainly reflect their expression stoichiometry. The overall ratio for rAAV from human embryonic kidney (HEK) cells is around 1:1:10.^9^, ^10^, ^11^, ^12^ Capsids from *Spodoptera frugiperda* (Sf9) cells using baculovirus expression appear to be less heterogeneous (i.e., they contain more VP3 and less VP1 and/or VP2).^13^ The genome is single-stranded DNA, 4.7 kb in the wild-type (WT) virus, with identical T-shaped inverted terminal repeats at the ends. Both + and – strands are packaged with equal frequency.^14^ Incomplete genomes can be packaged by both WT-AAV particles and rAAV vectors.^15^, ^16^, ^17^ Small DNA fragments can also be packaged as well as heterogeneous DNA.^18^, ^19^, ^20^, ^21^

Like all viruses, AAV must perform a thermodynamic balancing act.^22^ The capsid must be stable enough to protect the genome from the environment but also must be able to release its genetic payload at the right time and place for replication. The thermal stability of AAV particles and the structural changes induced by heating have been investigated in a number of studies.^23^, ^24^, ^25^, ^26^, ^27^, ^28^, ^29^, ^30^, ^31^ Several studies indicate that heating leads to externalization of the VP1 N termini.^23^^,^^24^^,^^28^ It is also well established that heating leads to genome release. Atomic force microscopy (AFM) has been used to probe structural changes and genome release in several studies where images show single-stranded DNA (ssDNA) extruding from the capsid.^26^^,^^30^ An inverse correlation between the packaged genome length and the temperature needed to induce uncoating was noted.^26^ Measurements using differential scanning fluorimetry (DSF) and differential scanning calorimetry (DSC) show serotype melting temperatures that vary by more than 20°C, with rAAV2 being the least stable and rAAV5 being the most.^28^ The transgene (luciferase) had only a minor effect, with the melting temperatures of empty and full particles differing by 1°C to 2°C at most.^29^ The nature of the buffer had a significant effect on the melting temperatures of AAV2 and AAV3, but for other serotypes studied, the effect was modest (a few degrees). It has been suggested that the thermal stability measured by DSF could be used as a determinant of AAV serotype identity.^29^

In this study, we have used charge detection mass spectrometry (CDMS) to investigate thermally induced transitions for rAAV8 particles with a variety of genome of interest (GOI) lengths from 2,219 to 4,844 nt. CDMS is a single-particle technique where the masses of individual ions are measured directly. It allows accurate mass distributions to be recorded for highly heterogeneous samples that are beyond the capabilities of conventional MS.^32^, ^33^, ^34^, ^35^, ^36^, ^37^ In CDMS, the *m*/*z* ratio and charge are determined simultaneously for each ion and then multiplied to give their masses. Masses measured for thousands of ions are binned into a mass distribution. In conventional MS, where just the *m*/*z* ratio is measured, the ion’s charge must be deduced from the *m*/*z* spectrum. However, when the sample has a heterogeneous mass distribution, there is a large number of overlapping *m*/*z* peaks in the *m*/*z* spectrum, and the charge cannot be determined. Heterogeneity increases with size, and conventional MS usually cannot determine masses for ions larger than around a 1 MDa without prior knowledge of the mass. CDMS has been used to measure molecular weights for species well over 100 MDa, such as adenovirus.^38^

CDMS has previously been used to investigate changes induced by incubation of AAV8 vectors at elevated temperatures, and it was found that incubation significantly narrowed the peaks attributed to both empty and full particles.^20^ This observation was attributed to the release of small DNA fragments. Marty and coworkers^39^ have also recently reported some preliminary results on heating of AAV vectors using a related approach, Orbitrap individual ion MS (I^2^MS).^40^, ^41^, ^42^, ^43^, ^44^

## Results

**Figure 1:**
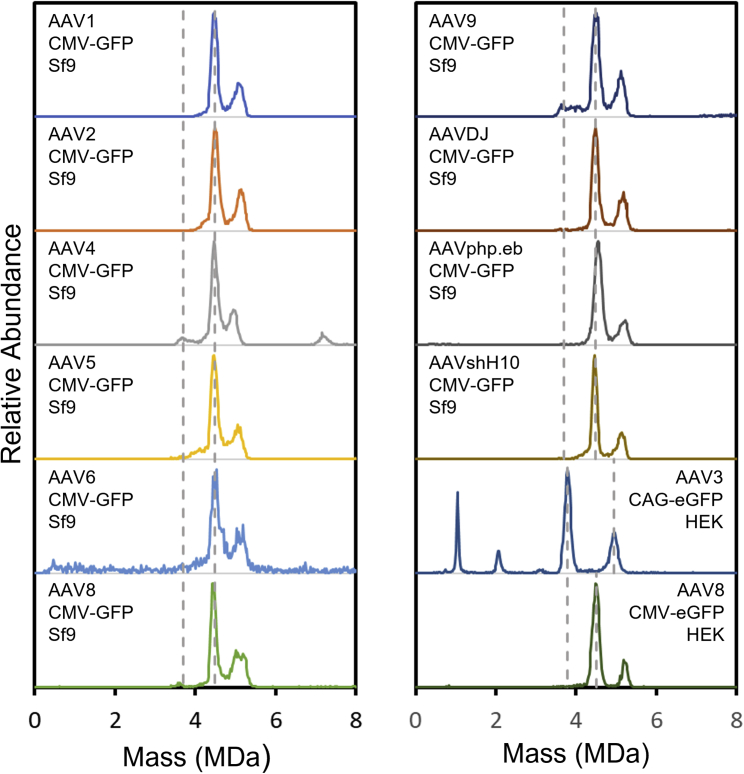
AAV serotype study CDMS mass distributions recorded for AAV1, AAV2, AAV4, AAV5, AAV6, AAV8, AAV9, AAVDJ, AAVphp.eb, and AAVshH10 with a CMV-GFP genome prepared in Sf9 cells and for AAV3-CAG-EGFP and AAV8-CMV-EGFP prepared in HEK cells. Dashed gray lines show the expected locations of the empty and full particles for each serotype and genome.

Previously, CDMS has been used to characterize empty-full ratios and the mass distributions for the packaged DNA, including partially filled and overpackaged particles.^17^^,^^20^ CDMS only requires 10–20 uL of material at >5 × 10^11^ particles/mL, and a spectrum can typically be collected in 25–60 min (depending on the signal intensity, which, in turn, depends on the sample concentration). Measurements have been performed for a wide variety of serotypes. As a demonstration, Figure 1 shows CDMS mass distributions recorded for AAV1, AAV2, AAV4, AAV5, AAV6, AAV8, AAV9, AAVDJ, AAVphp.eb, and AAVshH10 with a cytomegalovirus (CMV)-GFP genome prepared in Sf9 cells and for AAV3-CAG-EGFP and AAV8-CMV-EGFP prepared in HEK cells. Dashed gray lines show the expected location of the empty and full particles for each serotype and genome. Except for AAV3-CAG-EGFP, the dominant peak is due to the full particle, and the intensity at the expected mass of the empty is small. In addition to the dominant peak located at the expected mass of the full particle with the GOI, there is another smaller peak at a higher mass (around 5 MDa) for all spectra except AAV3-CAG-EGFP. The CMV-GFP and CMV-EGFP genomes are much smaller than the AAV packaging capacity. AAV particles that have packaged DNA to the packaging capacity are expected to have a mass around of 5 MDa, which corresponds to the mass of the smaller peak mentioned above. This peak has been attributed mainly to the packaging of a single strand of heterogeneous DNA from the host or plasmid.^20^ These results suggest that packaging of heterogeneous DNA occurs for samples prepared in HEK cells with roughly the same frequency as those prepared in Sf9 cells.^20^ The peak in the AAV4 spectrum at 7.2 MDa is at a mass slightly lower than expected for a dimer of the empty capsids, so it probably results from another impurity. The spectrum for AAV3-CAG-EGFP shows a sequence of peaks at 1.03, 2.06, and 3.09 MDa. These probably result from aggregates of an impurity with a molecular weight (MW) around 1.03 MDa. Finally, the AAV6 spectrum in Figure 1 shows a significant background signal at all masses. This probably results from disassembly and non-specific aggregation of the disassembly products. It appears that this AAV6 sample is particularly sensitive to freeze-thaw cycles, and after a second freeze-thaw cycle, peaks due to the full and overpackaged particles were no longer apparent in the spectrum.

**Figure 2:**
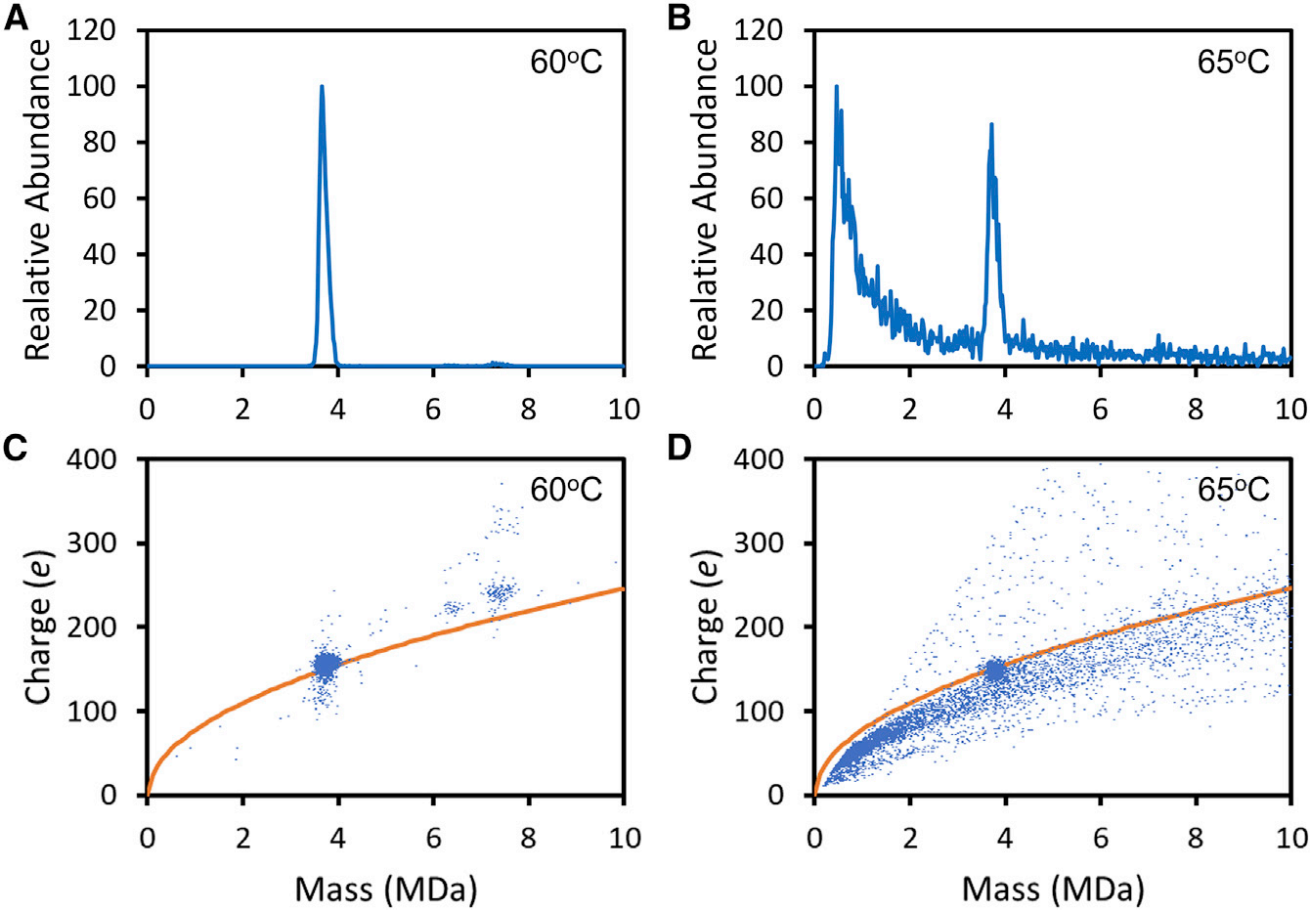
Incubation of empty AAV8 (A) CDMS mass distribution for empty AAV8 after incubation at 60°C for 15 min. (B) Mass distribution after incubation at 65°C for 15 min. (C and D) Charge versus mass scatter plots for the results shown in (A) and (B), respectively. The charge is plotted in units of elementary charge (e). The orange lines show the Rayleigh charge limit for a spherical object (see text).

Figures 2A and 2B show CDMS mass distributions measured for an empty rAAV8 capsids after being incubated for 15 min at 60°C and 65°C, respectively. The main peak in the spectrum at around 3.7 MDa is attributed to the empty capsid. Measurements were also made for incubation temperatures between 45°C and 55°C in 5°C increments (data not shown). The spectra measured between 45°C and 60°C are not significantly different from those measured for unheated samples. As the incubation temperature is raised to 60°C, the 3.7 MDa peak becomes slightly narrower and shifts to a slightly lower mass. These changes are probably due to the loss of small heterogeneous DNA fragments as the samples are heated and have been discussed elsewhere.^20^ Incubation also appears to diminish the small amount of multimers present at masses between 6 and 8 MDa to the point where they are barely visible in the 60°C mass spectrum in Figure 2A.

While incubation to 60°C causes only minor charges to the mass spectrum, dramatic changes occur for incubation at 65°C (see Figure 2B). The main peak at around 3.7 MDa is substantially diminished, and there is a broad distribution below the mass of the main peak and a high-mass tail that extends to beyond 50 MDa. The ions with masses below the main peak are attributed to capsid disassembly products, and the high-mass tail presumably results from their aggregation.

Figures 2C and 2D show charge versus mass scatter plots measured after incubation at 60°C and 65°C. Each point in the plots represents the measurement for a single ion. The 60°C scatter plot is like those measured at room temperature and lower incubation temperatures (data not shown). The tight cluster of ions centered on a mass of around 3.7 MDa and a charge of around 160 elementary charges (e) corresponds to the main peak in the mass distribution. There are also clusters with a few ions at higher mass (6–8 MDa) and charge that result from multimers.

Correlating mass and charge can provide information about the structure.^45^ Large ions generated by electrospray are thought to be produced by the charge residue mechanism,^46^^,^^47^ where a water droplet deposits its charge on the analyte as it evaporates away. The maximum charge deposited on a spherical ion can be predicted from the Rayleigh charge limit for a water droplet^48^ with the same diameter as the analyte: *z*_*R*_ = 0.778 m^1/2^. Compact spherical ions are expected to have charges that are 70%–100% of the Rayleigh limit. Charges significantly above the Rayleigh limit indicate an elongated structure or a structure with a larger diameter that can accommodate more charge. The orange lines in Figures 2C and 2D show the Rayleigh limit. The lines pass through the clusters at 3.7 MDa. These ions are expected to have the geometry of a hollow spherical shell. Most of the ions in Figure 2D fall below the line. Their lower charge suggests that they have more compact, near-spherical geometries.

**Figure 3:**
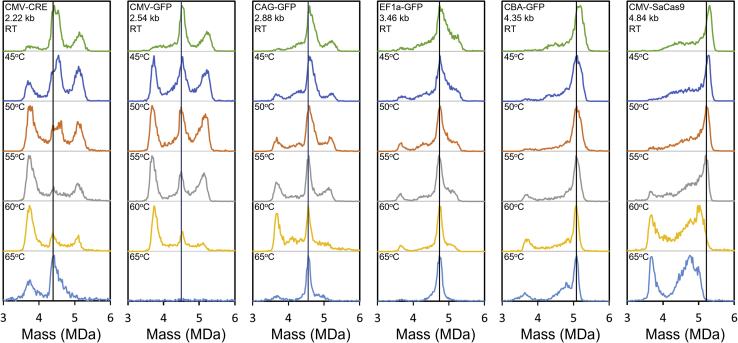
CDMS mass distributions measured for incubation of AAV8 vectors Results are shown for six different genomes ranging in size from 2.2 to 4.84 kb and for incubation temperatures of 45°C, 50°C, 55°C, 60°C, and 65°C. In all cases, the samples were incubated for 15 min. The top spectrum in each column (labeled RT) was measured without incubation. The black vertical lines are guides to show how the peak at close to the mass expected for the vector with a full genome varies with temperature.

Figure 3 shows CDMS mass distributions recorded for rAAV8 vectors as a function of incubation temperature. Results are shown for six different rAAV8 vectors with GOIs ranging in size from 2.22 to 4.84 kb. Table 1 shows a summary of the GOIs employed along with their lengths and sequence masses. The top spectrum for each vector in Figure 3 was recorded without incubation. The spectra below were recorded at incubation temperatures of 45°C–65°C in 5°C increments. Each spectrum contains over 10,000 single-ion events except for CVM-GFP at 65°C, where the signal was low. The black vertical lines in Figure 3 are guides to show the temperature-dependent behavior of the peak close to the expected mass of the vector with a full genome. A remarkable diversity of behavior is evident in Figure 3. However, there are some unifying themes. The peak at close to the expected mass for the full genome narrows and shifts to slightly lower mass as the temperature is raised. This behavior is attributed to the loss of small heterogeneous DNA fragments or other species that are copackaged with the genome. The empty particle has a mass of around 3.7 MDa, and as the temperature is raised, the number of empty particles generally increases except at the highest temperature (65°C), where it decreases. This is in line with the results shown in Figure 2 where the empty particle disassembles at 65°C. The results for CMV-SaCas9, the largest genome studied, are an exception.

**Table 1:** Promoter and genome for the rAAV8 vectors used in the incubation studies

Promoter and genome	Number of bases	Expected mass (MDa)^a^
Empty	0	0
CMV-CRE	2219	0.683
CMV-GFP	2544	0.783
CAG-GFP	2876	0.886
EF1a-GFP	3458	1.065
CBA-GFP	4354	1.341
CMV-SaCas9	4844	1.492

a From average of ionized + and – strands.

The peak at just over 5 MDa for the vectors with the three smallest genomes (CMV-CRE, CMV-GFP, and CAG-GFP) and the high mass shoulder for vectors with the EF1a-GFP genome were found in previous studies to be mainly due to the packaging of a single strand of heterogeneous DNA up to the packaging capacity.^20^ As the temperature is raised, the abundance of this peak generally declines. For vectors with CMV-CRE and CMV-GFP genomes (the two smallest), there are substantial changes in the mass distributions when the samples are incubated to 45°C. Specifically, there is a large increase in the abundance of the peak at the mass expected for the empty particles.

In general, changes in the mass distributions occur at lower temperatures for vectors with smaller genomes. Thus, with an incubation temperature of 50°C, there are modest changes in the mass distributions for CBA-GFP and CMV-SaCas9 compared with the spectra for unincubated samples, but the mass distributions for CMV-CRE and CMV-GFP are significantly different, with a large increase in the abundance of empty particles at around 3.7 MDa. At 65°C for the CMV-GFP sample, there is little intensity left in the 3–6 MDa mass range. For CAG-GFP, EF1a-GFP, and CBA-GFP with an incubation temperature of 65°C, there are prominent peaks at the expected mass for particles with a full genome. However, for CMV-SaCas9, the peak that was at the expected mass for the vector with a full genome disappears for incubation temperatures above 55°C. The CMV-SaCas9 genome is slightly longer than the WT (4.84 versus 4.7 kb), so this may be the reason for its reduced stability.

**Figure 4:**
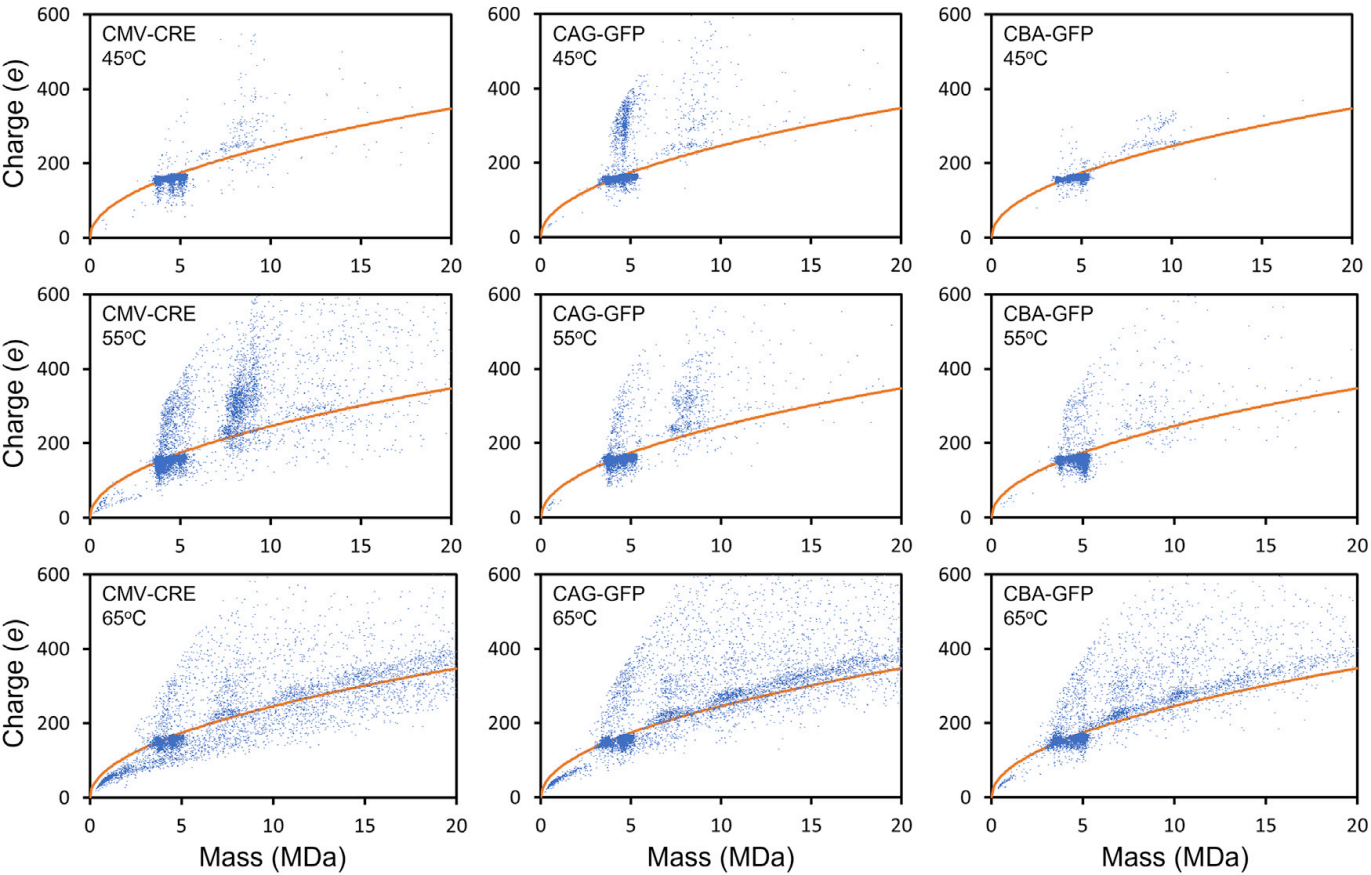
Representative charge versus mass scatter plots for incubation of AAV8 vectors Results are shown for three different genomes (CMV-CRE, CAG-GFP, and CBA-GFP) for incubation temperatures of 45°C, 55°C, and 65°C. In all cases, the samples were incubated for 15 min. The orange lines show the Rayleigh charge limit for a spherical object (see text).

Figure 4 shows some representative charge versus mass scatter plots. The orange lines in the scatter plots show the Rayleigh charge limit. Charges substantially above this line suggest the ions have larger or elongated structures. It is evident that streaks of highly charged ions form as the incubation temperature is raised. In the case of CAG-GFP, a streak is evident at 45°C. For the other vectors, the streaks first emerge at a higher temperature.

**Figure 5:**
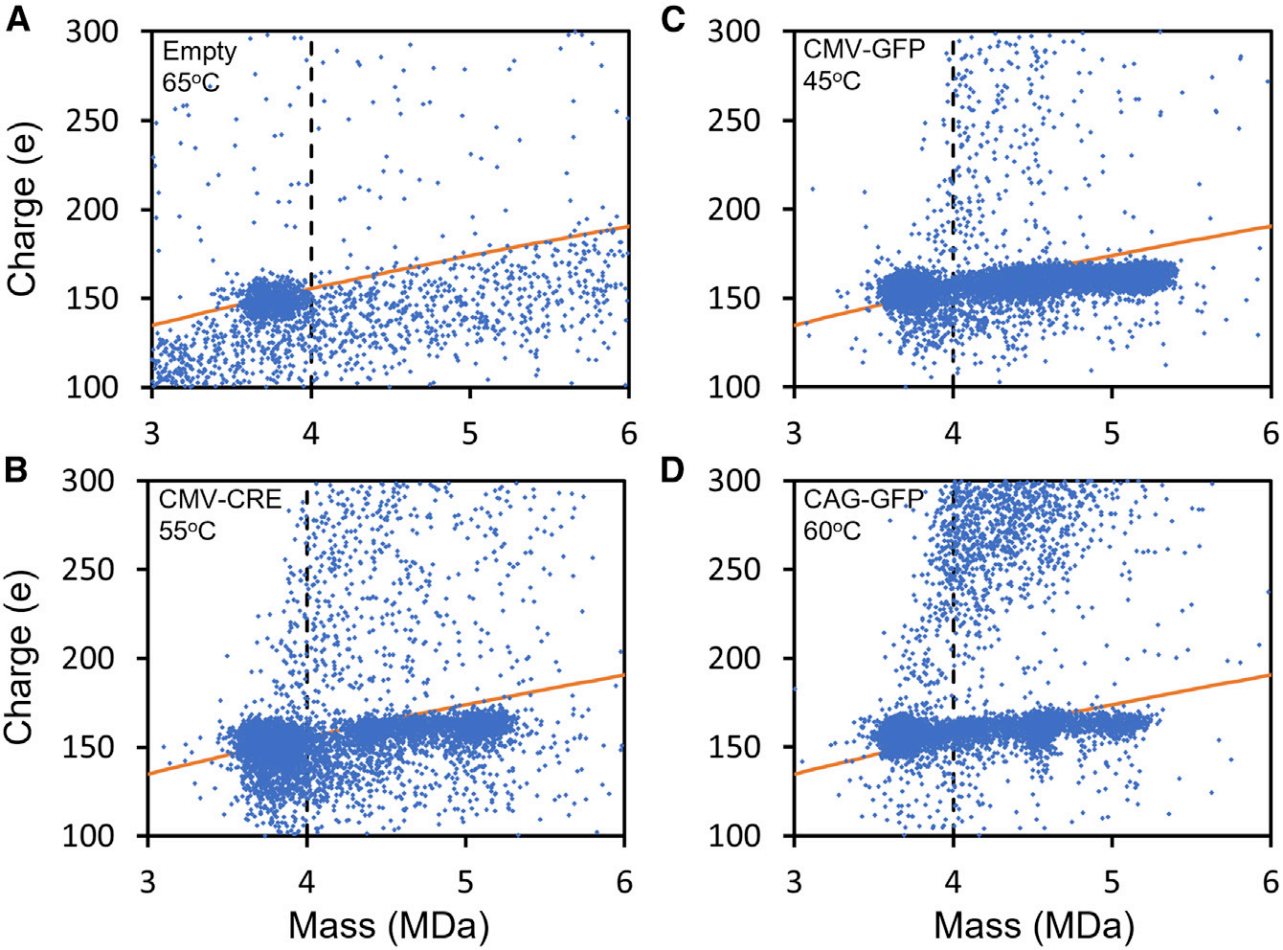
Zoomed-in views of some representative charge versus mass scatter plots for incubation of AAV8 (A) Results for empty AAV8. The dashed vertical line shows the high-mass extent of the empty capsid. The orange line shows the Rayleigh charge limit for a spherical object (see text). (B–D) Portions of the scatter plots for CMV-CRE at 55°C, CMV-GFP at 45°C, and CAG-GFP at 60°C.

Figure 5 shows zoomed-in views of portions of the charge versus mass scatter plots. For comparison, Figure 5A shows a view of a portion of the scatter plot for the empty particle after incubation at 65°C. The dashed vertical line is a guide that shows the high-mass extent of the empty particle. Note that most of the ions lie below the Rayleigh charge limit (orange line), indicating relatively compact geometries. There is no streak of highly charged ions for the empty particle. Figure 5B shows a zoomed-in view of the charge versus mass scatter plot for CMV-CRE at 55°C. It is evident that most of the high-charge streak occurs for masses that are greater than the maximum extent of the empty capsid (the vertical dashed line). Similar behavior is found for the other examples shown in Figures 5C and 5D. In all cases, the high-charge streak is populated mainly by ions with masses that are larger than the mass of the empty particle. This observation, and the absence of a streak for the empty particles, leads us to hypothesize that the high-charge streak results from DNA extruding from the capsid. From the results in Figure 3, the intensity at the mass attributed to the empty particle (around 3.7 MDa) initially increases as the incubation temperature is raised, indicating that DNA is being lost from some of the vectors.

In addition to the high-charge streaks, another feature of the charge versus mass scatter plots in Figure 4 is the appearance of ions with masses greater than the mass of the full capsid (around 5 MDa) as the temperature is raised. These higher-mass ions probably result from aggregation. Clusters of ions at multiples of the unaggregated particle mass are evident at 65°C for all the genomes in Figure 4. Particles with masses at multiples of the empty capsid are the most abundant. Full particles show a lower propensity to aggregate. Note that many of the dimers (and the higher-order multimers) have charges that lie above the Rayleigh limit (orange lines in the figure), which is consistent with them having dimer-like geometries rather than a spherical geometry. Some of the dimers have high charges substantially above the Rayleigh limit. See, for example, the results in Figure 4 for CMV-CRE at 55°C. The highly charged ions could result from the aggregation of a particle with extruded DNA with an empty particle.

**Figure 6:**
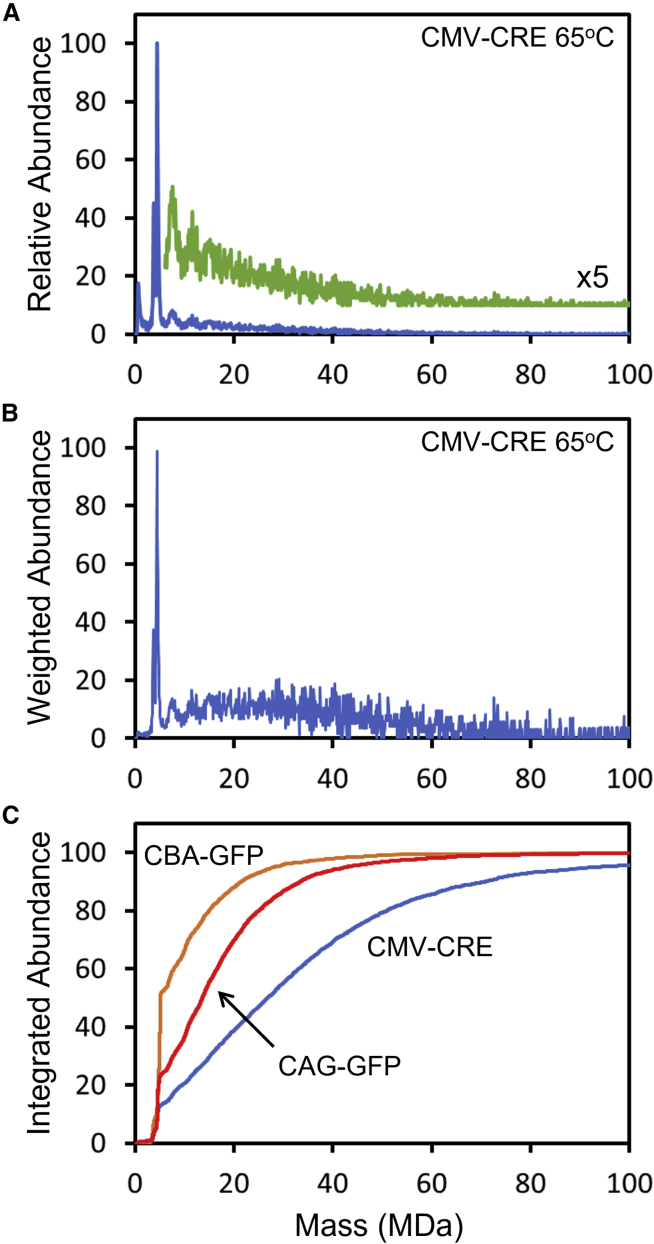
Mass distributions for AAV8 aggregates (A) Mass distribution for CMV-CRE at 65°C. (B) A plot of the mass weighted abundance (the intensity at each mass times the mass) against the mass for CMV-CRE at 65°C. (C) The integrated abundance (the integral of the mass weighted abundance from zero to a given mass) plotted against the mass. Results are shown for three different genomes (CMV-CRE, CAG-GFP, and CBA-GFP) for an incubation temperature of 65°C.

Figure 6A shows the mass distribution measured for CMV-CRE at 65°C. In this spectrum, aggregates extend to beyond 100 MDa. This way of presenting the data is misleading because it underestimates the contribution of particles in multimers. An unaggregated capsid receives the same weight as an aggregate of 20 capsids. Figure 6B shows an alternative way of representing the aggregate distribution where the weighted abundance is plotted against the mass. The weighted abundance is obtained by multiplying the relative abundance at each mass by the mass. It is evident from this plot that only a small fraction of the total mass remains in the unaggregated monomer, indicating a substantial drop in the capsid titer. This is made clear in Figure 6C, which shows a plot of the integrated weighted abundance against the mass. The blue line shows the results for CMV-CRE at 65°C. According to this plot, only around 13% of the monomer remains unaggregated, and around 5% of mass is in aggregates with masses larger than 100 MDa. The red line in Figure 6C shows results for CAG-GFP at 65°C. In this case, there is less aggregation than for CMV-CRE; around 24% remains unaggregated, and most of the aggregates are under 60 MDa. For CBA-GFP at 65°C, 52% of the monomer remains, and most of the aggregates are under 50 MDa. The general trend, evident in Figure 6C, is that there is less aggregation as the genome size increases. This observation is probably related to the longer genomes stabilizing the capsids.

**Figure 7:**
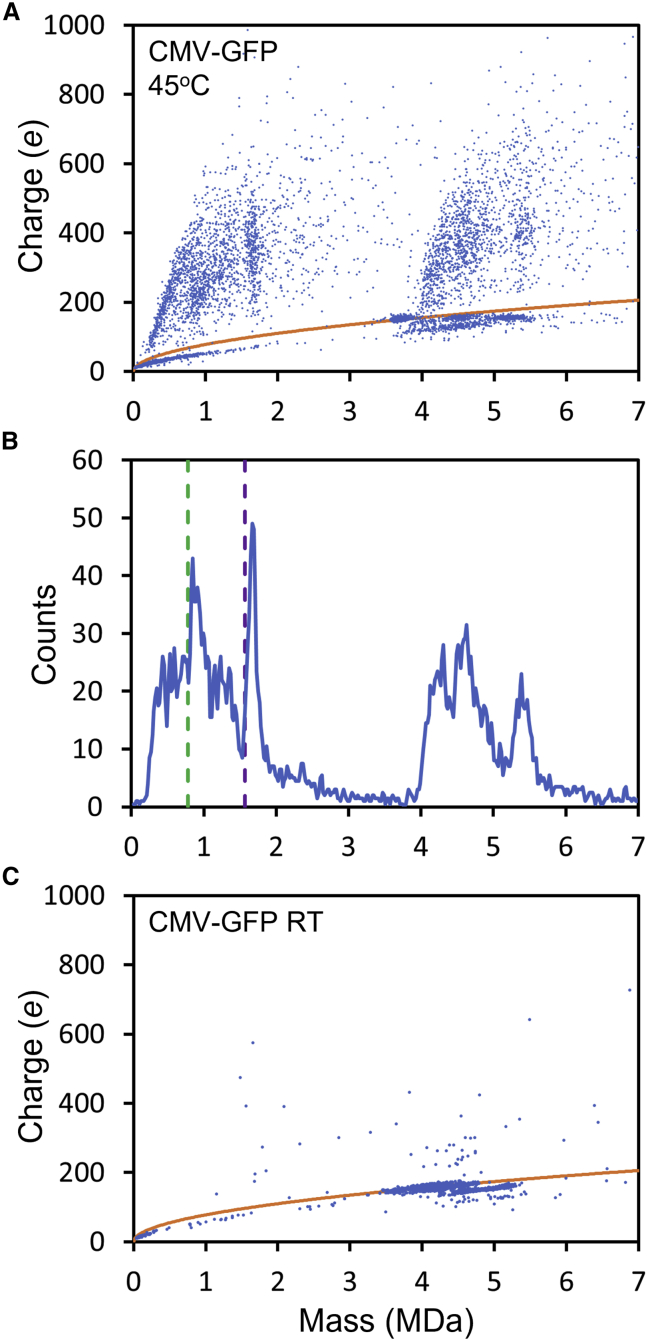
CDMS measurements showing extracted DNA (A) A charge versus mass scatter plot measured for AAV8-CMV-GFP incubated at 45°C using conditions optimized to transmit highly charged DNA ions. The orange lines show the Rayleigh charge limit for a spherical object (see text). (B) Mass distribution for ions in (A) with charges more than 10% over the Rayleigh charge limit. The green dashed lines show the expected masses for single-stranded GOI, and the purple dashed lines show the expected masses for double-stranded GOI. (C) Charge versus mass scatter plot for unincubated AAV8-CMV-GFP measured using conditions optimized to transmit highly charged DNA ions.

The high-charge streaks in Figure 4 were attributed to DNA extruding from the AAV capsids. As the incubation temperature is increased, there is often an increase in the abundance of empty particles, suggesting that the DNA is eventually lost from the capsids. Previous studies have shown that isolated DNA ions can be highly charged because they can adopt elongated geometries.^32^^,^^49^^,^^50^ To detect the extruded DNA, we found it was necessary to optimize the conditions to transmit highly charged ions. Figure 7A shows a charge versus mass scatter plot for CMV-GFP at 45°C with conditions optimized to transmit DNA ions. There are many highly charged, low-mass ions in this plot, including a prominent vertical streak at around 1.7 MDa with charges that span from around 200 to 500 e.

The orange line in Figure 7A shows the Rayleigh charge limit for a spherical ion. While many ions have charges above the Rayleigh limit, there is a streak of ions between 0 and 2 MDa with charges below the Rayleigh charge limit. While a small feature in the scatter plot, this low-charge streak contains a significant fraction of the ions in the plot. We attribute these ions to compact protein aggregates. Figure 7B shows the mass distribution for ions in Figure 7A with charges that are larger than 110% of the Rayleigh charge limit. This provides a mass distribution for the highly charged ions while discarding ions with lower charge, such as the protein aggregates mentioned above.

There are several features in the mass distribution in Figure 7B. The signal between 4 and 6.5 MDa is attributed to AAV with extruded DNA discussed above. There are subpopulations in this mass range that can be correlated with subpopulations evident in Figure 3. There are also subpopulations below 2 MDa. Notably, a sharp peak centered on 1.66 MDa corresponds to the vertical streak at the same mass in Figure 7A. AAV packages a ssDNA genome. Both + and – strands are packaged with equal frequency,^14^ and when released, the + and – strands can base pair in solution. The dashed purple line in Figure 7B shows the expected mass of the double-stranded (ds) CMV-GFP (1.566 MDa). The peak in Figure 7B is around 90 kDa higher in mass than expected for the base-paired genome. This excess mass can be partly attributed to counter ions and ions associated with the net charge. At least some of the backbone phosphate groups of DNA are negatively charged in solution and must have counter ions in the gas phase. In addition, the DNA ions are positively charged with a net charge of 200–500 e. If the counter ions are NH_4_^+^ (the ions are electrosprayed from an ammonium acetate solution) and the overall charge is provided by attached NH_4_^+^ ions, a maximum additional mass of 101 kDa is expected. On the other hand, if all the backbone phosphates are unionized (i.e., H^+^ counter ion) and the net charge is provided by protonation of the DNA bases, the minimum additional mass is 5 kDa (for a net charge of 200 e) The measured excess mass (90 kDa) falls within the expected range (5–110 kDa), so we assign the peak at 1.66 MDa to dsGOI. The expected mass of the ssGOI is shown in Figure 7B by the dashed green line. There is a peak at a slightly higher mass than the dashed green line that is probably due to the ssGOI. It is broader than the peak attributed to the dsDNA GOI and sits on a higher background. Some of the background could be due to DNA-protein complexes.

To confirm that the DNA evident in Figure 7B results from incubation of AAV particles containing a CMV-GFP genome, Figure 7C shows a charge versus mass scatter plot for unincubated particles, measured using conditions identical to those used for Figure 7A (i.e., optimized to transmit highly charged ions). There are few highly charged ions, indicating that the DNA ions observed in Figures 7A and 7B result from incubation causing the particles to release the genome.

## Discussion

In the results presented here, disassembly of the empty capsid is almost complete after incubation at 65°C for 15 min. Since there is no measurable disassembly at 60°C, the disassembly transition for the empty capsid is sharp, suggesting a cooperative process analogous to a phase transition. On the other hand, for particles with a genome, genome loss occurs over a broad temperature range.

Several different types of disassembly behavior are evident in the results presented here. For short genomes (CMV-CRE and CMV-GFP), genome loss starts at a low temperature (as low as 45°C for CMV-GFP), leading to a substantial increase in the abundance of empty particles in the mass distributions at low incubation temperatures. For these vectors, after incubation at 65°C, most of the capsids, both empty and full, have disassembled into small fragments that then form aggregates with masses that extend to beyond 100 MDa. For the larger genomes (EF1a-GFP and CBA-GFP) that have lengths less than the WT genome (4.7 kb), the formation of empty particles is delayed to higher incubation temperatures, and the relative abundances of the empty particles formed are much smaller than for the particles with smaller GOIs. At 65°C, the main species remaining in the mass distribution is the capsid with the GOI. In these cases, the full particles survive to a higher temperature than the empty particles, as the longer GOIs presumably stabilize the capsids. For the CMV-SaCas9 genome (which is longer than the WT genome), the peak corresponding to the particle with the GOI disappears at around 60°C, and there is a corresponding increase in the relative abundance of the empty particles. Apparently, having a genome longer than the WT is destabilizing and leads to genome loss at a significantly lower temperature, even if the genome is only slightly longer than the WT (4.84 versus 4.7 kb). Note that intensity for masses corresponding to partially filled capsids (around 4.8 MDa) persist to 65°C for the longer genomes.

For short genomes, genome release and capsid disassembly are clearly well-separated events, with genome release occurring at a significantly lower temperature than that required for disassembly of the empty capsid (60°C–65°C). As the genome length increases, the temperature required for genome release increases and eventually exceeds the temperature for disassembly of the empty capsid. At this point, genome release and capsid disassembly may become contemporaneous. However, as the genome exceeds the WT length, the trend reverses, and genome loss again occurs at a lower temperature than disassembly of the empty capsid.

The peaks in Figure 3 for the smaller genomes at around 5.1 MDa have been attributed to packaging of heterogeneous DNA from host cells or plasmids. Note that this DNA does not remain internalized for temperatures up to 65°C. Some of this DNA could exceed the WT length, and this could be responsible for its loss at lower temperatures than the longer genomes (i.e., it could be behaving like the CMV-SaCas9 genome). However, some of this heterogeneous DNA is similar in length to the CBA-GFP genome; the fact that this DNA is released at a lower temperature suggests that some feature of the GOI stabilizes it inside the capsid and inhibits its release.

In previous work, we explored the idea of determining the genome mass from the difference between measured masses of the empty and full particles. We found that the most accurate values were obtained by incubating the particles to release small DNA fragments that are packaged in both the empty and full particles. The results presented here suggest that direct measurement of the extracted genome mass either as a single strand or after base pairing in solution may be a better approach. We have measured masses of extracted DNA from a variety of AAV vectors and will report on the results elsewhere.

## Materials and methods

### AAV samples

The empty AAV8 and the AAV8 vectors used in the incubation studies were purchased from Virovek, where they were prepared using baculovirus expression in Sf9 cells and purified using ultracentrifugation with a CsCl gradient. The samples used in the serotype study (Figure 1) with a CMV-GFP genome were also purchased from Virovek and prepared using Sf9 cells. The AAV3-CAG-EGFP and AAV8-CMV-EGFP samples for the serotype study were purchased from Vector Biolabs, where they were prepared in HEK cells. Upon receipt, the samples were aliquoted and stored at −80°C. Stored aliquots were thawed at 4°C. The aliquots were incubated in a water bath (set to the desired temperature) for 15 min and then quenched on ice for 1 min. Prior to electrospray, 10 μL volumes were exchanged into 200 mM ammonium acetate (Honeywell 631-31-8) solution (pH 7.3) using Micro Bio-Spin columns (Bio-Rad, 7326221). The samples were electrosprayed at room temperature. In some cases, incubation was performed both before and after buffer exchange, and the results were similar. This is consistent with the results of Bennett et al.,^29^ who reported that the melting temperature determined by DSF for AAV8 in different buffers varied by only a few degrees.

### CDMS

CDMS is a single-particle approach where the *m*/*z* ratio and charge are simultaneously measured for each ion. The mass of each ion is then obtained from the product of its *m*/*z* and charge. Mass measurements are performed for thousands of ions, and then the results were binned to yield a mass distribution. The measurements reported here were performed on our updated prototype CDMS instrument.^51^, ^52^, ^53^ The ions are trapped in an electrostatic linear ion trap and oscillate back and forth through a detection cylinder. When an ion enters the detection cylinder, it induces a charge that is detected by a charge-sensitive amplifier. The cylinder is long enough that the full ion charge can be measured, allowing the charge to be determined with enough accuracy to have well-resolved charge states in the charge spectrum.

## Data availability

The datasets generated during this study consists of tables of *m/z* and charge values measured by CDMS for individual ions. These datasets are available from the corresponding author on reasonable request.
